# A method for reporting and classifying acute infectious diseases in a prospective study of young children: TEDDY

**DOI:** 10.1186/s12887-015-0333-8

**Published:** 2015-03-20

**Authors:** Maria Lönnrot, Kristian Lynch, Helena Elding Larsson, Åke Lernmark, Marian Rewers, William Hagopian, Jin-Xiong She, Olli Simell, Anette-G Ziegler, Beena Akolkar, Jeffrey Krischer, Heikki Hyöty, The TEDDY study group

**Affiliations:** University of Tampere, Tampere, Finland; Seinäjoki Central Hospital, Seinäjoki, Finland; University of South Florida, Tampa, USA; Lund University, Malmö, Sweden; Barbara Davis Center for Childhood Diabetes, Denver, CO USA; Pacific Northwest Diabetes Research Institute, Seattle, USA; Georgia Regents University, Augusta, USA; University of Turku, Turku, Finland; Institute of Diabetes Research, Helmholtz Zentrum München, and Klinikum rechts der Isar, Technische Universität München, and Forschergruppe Diabetes e.V, Neuherberg, Germany; NIH, Bethesda, USA; Fimlab Laboratories, Pirkanmaa Hospital District, Tampere, Finland

**Keywords:** Childhood infections, Prospective study, Type 1 diabetes

## Abstract

**Background:**

Early childhood environmental exposures, possibly infections, may be responsible for triggering islet autoimmunity and progression to type 1 diabetes (T1D). The Environmental Determinants of Diabetes in the Young (TEDDY) follows children with increased HLA-related genetic risk for future T1D. TEDDY asks parents to prospectively record the child’s infections using a diary book. The present paper shows how these large amounts of partially structured data were reduced into quantitative data-sets and further categorized into system-specific infectious disease episodes. The numbers and frequencies of acute infections and infectious episodes are shown.

**Methods:**

Study subjects (n = 3463) included children who had attended study visits every three months from age 3 months to 4 years, without missing two or more consecutive visits during the follow-up. Parents recorded illnesses prospectively in a TEDDY Book at home. The data were entered into the study database during study visits using ICD-10 codes by a research nurse. TEDDY investigators grouped ICD-10 codes and fever reports into infectious disease entities and further arranged them into four main categories of infectious episodes: respiratory, gastrointestinal, other, and unknown febrile episodes. Incidence rate of infections was modeled as function of gender, HLA-DQ genetic risk group and study center using the Poisson regression.

**Results:**

A total of 113,884 ICD-10 code reports for infectious diseases recorded in the database were reduced to 71,578 infectious episodes, including 74.0% respiratory, 13.1% gastrointestinal, 5.7% other infectious episodes and 7.2% febrile episodes. Respiratory and gastrointestinal infectious episodes were more frequent during winter. Infectious episode rates peaked at 6 months and began declining after 18 months of age. The overall infectious episode rate was 5.2 episodes per person-year and varied significantly by country of residence, sex and HLA genotype.

**Conclusions:**

The data reduction and categorization process developed by TEDDY enables analysis of single infectious agents as well as larger arrays of infectious agents or clinical disease entities. The preliminary descriptive analyses of the incidence of infections among TEDDY participants younger than 4 years fits well with general knowledge of infectious disease epidemiology. This protocol can be used as a template in forthcoming time-dependent TEDDY analyses and in other epidemiological studies.

**Electronic supplementary material:**

The online version of this article (doi:10.1186/s12887-015-0333-8) contains supplementary material, which is available to authorized users.

## Background

The cause of type 1 diabetes (T1D) is widely considered to involve the interaction of environmental exposures with a common genetic predisposition leading to the autoimmune destruction of the insulin-secreting beta cells in the pancreas. However, the specific causes and causal pathways remain largely unknown. Understanding the role of the environment in the etiology and pathogenesis of T1D could greatly improve the ability to prevent the disease. Infections are among the environmental candidates suggested to play a role in T1D.

The Environmental Determinants of Diabetes in the Young (TEDDY) study evaluates multiple environmental exposures in a prospective follow-up of children with increased genetic risk for T1D. The specific aim of TEDDY is to identify and characterize the environmental factors that may trigger T1D-related autoimmune process and/or promote progression to clinical T1D [1]. In TEDDY, prospective data on infectious exposures among study participants are being collected though a reporting system whereby parents monitor and record illnesses between clinical visits. In addition, biological samples such as plasma, stool and nasal swab samples are being collected at regular intervals. The present study focuses on the parental reports of acute infectious illnesses.

Infections may either increase or decrease the risk of T1D, and their mode of action may depend on several factors, such as 1) the type of infection, e.g. only certain microbes or only infections in certain anatomical location may be related to T1D; 2) the quantity of infections, e.g. low or high overall rate of infections, 3) timing of infections, e.g. autoimmunity might be triggered by an infection only during certain period of age, 4) febrile host response to infection 5) genetic background of the infected host and 6) a combination of any of these. Due to this possible complexity a system enabling a time dependent analysis of infectious disease data at broad and specific levels is needed. We therefore sought to organize the complex data from parental reports so that single infectious agents as well as broader categories of infectious agents or clinical disease entities could be simultaneously analyzed.

Multiple symptoms and signs in various organs and anatomical sites are often seen during the course of a single infection [2,3]. This can generate several symptom reports and /or diagnosis reports during one infection. These may occur simultaneously on a same day, or sequentially with some days in between. If each of these reports is regarded as a separate infection, the total number of microbial exposures becomes overestimated. In order to get closer to the real number of exposures we designed an infectious episode approach in which infectious disease reports representing presumably the same infection are merged together into an infectious episode.

In epidemiological research it is common that associations observed between reported data and a disease provide the vital first steps towards generating research hypothesis. However, when disease incidence is low and reported environmental exposures are hypothesized to modulate disease risk, it becomes highly relevant to understand how reported data is processed. In the present paper we describe the infectious disease report processing in great detail to enable validation of results across studies, which will subsequently generate valid research hypotheses for designing analysis of the costly biological samples.

The aim of the present paper is 1) to describe how parental reports of children’s infectious diseases were processed in TEDDY study, 2) to describe the scheme for creating infectious episodes and 3) to show the numbers and frequencies of acute infectious diseases and infectious episodes during first four years of life of TEDDY subjects.

## Methods

### Study subjects

TEDDY is a multicenter prospective follow-up study of children enrolled in one of six clinical centers in Finland, Germany, Sweden and the United States (Colorado, Florida/Georgia and Washington). Newborns invited into TEDDY study belong to either general population (GP) or are first-degree relatives (FDR) of a patient with T1D. The TEDDY cohort was identified and enrolled between December 2004 and February 2010 through genotyping newborns after receiving parents’ informed consent. Human leukocyte antigen (HLA) class II gene combinations associated with an increased risk for T1D were used in genotyping and children with increased HLA-related risk were invited to participate the follow-up as previously described [4]. The average absolute risk for T1D is 4.0% in GP and 13.3% in FDR as compared to up to 1% absolute risk in background population of TEDDY countries (T1D incidence is 30-50/100 000 in Scandinavia, 15-25/100 000 in the US and 5-15/100 000 in Eastern and Central Europe) [4]. As T1D is an endpoint in the TEDDY study, children who develop T1D are no longer followed in the study. Total number of subjects in the TEDDY cohort is 8,677. The study protocol has been described in detail previously [4] and the TEDDY Study Group is described in Additional file 1. Written informed consents were obtained for all study participants from a parent or primary caretaker, separately, for genetic screening and participation in prospective follow-up. The study was approved by local Institutional Review Boards and is monitored by External Advisory Board formed by the National Institutes of Health.

Subjects of the present study were identified among TEDDY cohort at the end of year 2012. We included all subjects who had attended follow-up visits for four years since birth, had not progressed to T1D, and had not missed two or more consecutive study visits. Total number of subjects in the present study is 3,463 (General Population n = 3,078, First-degree relatives n = 385), including subjects from all study sites: Finland n = 815, Germany n = 164, Sweden n = 1271, Colorado n = 545, Florida/Georgia n = 285 and Washington n = 383.

### Data collection

Enrollment and completion of the first study clinic visit occurs before the child reaches the age of 4.5 months. After the enrollment, the subject is expected to attend TEDDY follow-up visit every 3 month until age 4 years and every 6 months thereafter. Parents are asked to record immediately child’s environmental exposures (infections, medical conditions, food intake and life events) in a TEDDY diary book they keep at home and return to study nurse at each TEDDY visit. In case of illnesses parents fill in symptoms and/or diagnoses and the date when the illness first appeared in the TEDDY diary book. Fever can be reported as a symptom and/or as a “yes” –answer to a specific question asking if the child had fever, defined as temperature equal to or higher than 38°C or 101°F. This specific question was used to classify fever in more detail since systemic fever may alter the nature of immune response, which could influence the natural course of the autoimmune process leading to T1D. Parents return the diary book on each clinic visit when data is extracted by research nurses to the TEDDY central database. Clinic visits are scheduled every three months for the first four years of life and then biannually until age 15. The TEDDY book pages for illness reports for 0–2 and 2–5 year old children are shown as additional files (see Additional files 2 and 3).

### Data management

**Figure 1:**
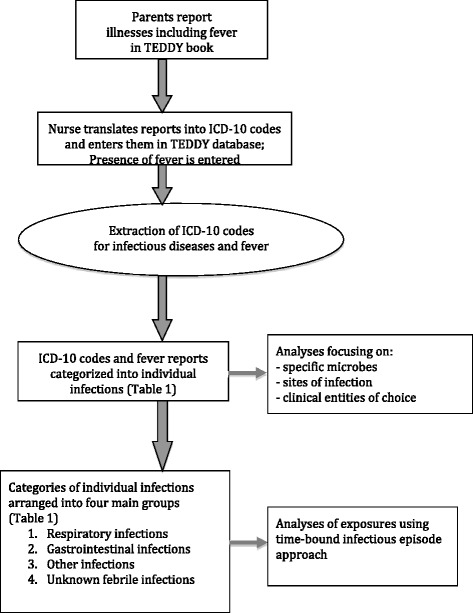
**Flow of infectious disease data recorded with TEDDY book.**

The flow of infectious disease data collection is shown in Figure 1. At each clinic visit, reported symptoms and diagnoses are translated into separate codes by a trained study nurse according to the current version of World Health Organization’s International Classification of Disease (ICD-10) system. ICD-10 codes with either three or four alphanumerical characters are used. Study doctor assists with coding on nurse’s request. Symptoms are always recorded and diagnoses are categorized according to whether it was set by a health-care professional or not. “Yes”-answers to the specific fever-question are extracted as such. The ICD-10 codes and “yes”-fever reports along with the date of first appearance of the illness are entered into TEDDY database by study nurse on each visit. Acute and chronic illnesses are entered into the database separately. Data extraction form for acute illnesses is shown as an additional file (see Additional file 4).

ICD-10 codes for infectious diseases and “yes”-fever reports were extracted from the database’s section of acute illnesses and grouped into categories of interest (see Additional file 5). The extracted ICD-10 codes can be used for infectious disease analyses as such, if one wants to evaluate codes for certain microbe/microbes or codes for certain clinical entity/entities.

An infectious episode approach was designed in order to reduce the possibility of overestimation of microbial exposure due to multiple symptom and/or diagnosis reports during a single microbial infection. For this approach, the ICD-10 code categories described in Additional file 5 were arranged into four main categories representing: respiratory infections, gastrointestinal infections, other infections and unknown febrile infections (Table 1). Also, time-limits for merging ICD-10 codes into time-bound infectious episodes are required. Definitions for merging codes are the following:

**Table 1:** **Categories of infections arranged into four infectious episode types**

**Infectious episode type**	**ICD-10 code categories (see Additional file** 5 **)**	**Number of infectious episodes (% of total 71578)**	**Percent of febrile infectious episodes**
**Respiratory**	2.1. Common cold	52965 (74.0)	41.5
	2.2. Laryngitis and tracheitis		
	2.3. Influenza		
	2.4. Enterovirus		
	2.15. Respiratory syncytial virus infection		
	3. TONSILLITIS OR STREPTOCOCCAL PHARYNGITIS		
	4. SINUSITIS		
	5. INFECTIONS OF MIDDLE EAR AND MASTOID PROCESS		
	6. BRONCHITIS AND LOWER RESPIRATORY INFECTIONS		
	11.1. Conjuctivitis		
	11.6b. Other bacterial diseases, not elsewhere classified (respiratory tract)		
	With any of the above:		
	1. FEVER		
	8. GASTROENTERITIS SYMPTOMS		
**Gastrointestinal**	7. INFECTIVE GASTROENTERIS	9391 (13.1)	36.6
	8. GASTROENTERITIS SYMPTOMS^a^		
	With any of the above		
	1. FEVER		
**Other**	2.5. Chicken pox/Varicella	4081 (5.7)	54.0
	2.6. Zoster		
	2.7. Erythema infectiosum [fifth disease, parvovirus]		
	2.8. Exanthema subitum		
	2.9. Measles		
	2.10. Mumps		
	2.11. Rubella		
	2.12. Herpes simplex virus infection		
	2.13. Other viral rash		
	2.14. Infectious mononucleosis		
	2.16. Viral infections of the central nervous system, not elsewhere classified		
	2.17. Other virus infections, not elsewhere classified		
	2.18. Viral warts and molluscum		
	9. PARASITES		
	10. MYCOSE		
	11.2 Infections of external ear		
	11.3. Urinary tract infections		
	11.4. Other genitourinary infections		
	11.5. Bacterial skin diseases		
	11.6a. Other bacterial diseases, not elsewhere classified (non-respiratory tract)		
	11.7. Diseases of oral cavity		
	11.8. Lymphadenitis		
	11.9. Infections related to pregnancy, childbirth and puerperium		
	11.10. Perinatal/neonatal infections		
	11.11. Other and unspecified infections		
	With any of the above		
	1. FEVER		
**Unknown febrile**	1. FEVER without any ICD-10 code reported within a week prior or after.	5141 (7.2)	100

^a^gastroenteritis symptom associated with respiratory illness was not considered gastrointestinal.

1. The first infectious episode category is created from ICD-10 codes regarded as respiratory infections. If codes belonging to this category were reported within the same week, i.e. with a maximum of five days in-between the reports, they were regarded as one respiratory infectious episode. Gastrointestinal symptoms are considered to occur frequently in young children with a respiratory infection and therefore gastrointestinal symptom codes connected with respiratory infection codes reported within same week were considered as part of the respiratory infectious episode. Any fever reports within same week with respiratory infection codes were considered as part of the respiratory infectious episode. Date of the episode was the date of the first ICD-10 code.
2. The second category of an infectious is created from ICD-10 codes regarded as gastrointestinal infections. If codes belonging to this category were reported within same week they were regarded as one gastrointestinal infectious episode. Any fever reports within the same week with gastrointestinal infection codes were considered as part of the gastrointestinal infectious episode. Date of the episode was the date of the first ICD-10 code.
3. Third category is reserved for other types of infections. ICD-10 codes in this category are each regarded as a separate infectious episode. Any fever reports within the same week with other infection codes were considered as part of the other infectious episode category. Date of the episode was the date of the ICD-10 code.
4. Fourth category is for unknown febrile infections. This category includes fever reports which have been entered into database solely, without any other ICD-10 code recorded at the same date, nor any ICD-10 code for infection recorded within a week before or after the fever report. Date of the episode was the date of the fever report.

### Statistical analyses

Using the date of an infectious episode to determine, the percent of subjects reporting an infectious episode was calculated every 3 months over the first 4 years of life. The rate of infections over the 4 years was modeled as function of gender, HLA risk group, being GP/FDR and study center using a multivariate Poisson regression model. For each categorical variable added into the model, Wald tests tested for an overall significant difference across subgroups. Results were presented as rate ratios (95%CI) or as a percent change in the rate. P-values less than 0.05 were considered statistically significant. Graphs were drawn using graphpad 5.0 and statistical analysis was performed using SAS 9.3.

## Results

Altogether 113,884 ICD-10 code reports for infectious diseases were extracted from the database of acute illnesses for the 3,463 children followed until 48 months of age. Of the reported ICD-10 code reports 54,216 were described with a fever. Additionally 285 fevers were reported with no ICD-10 code. Numbers of reports in each infectious disease category are shown in Additional file 5.

**Figure 2:**
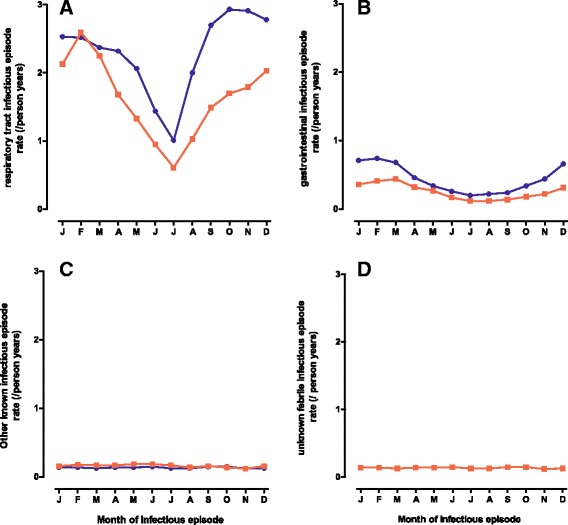
**Infectious episode rates by season.** Respiratory **(A, n = 52 965** **)**, Gastrointestinal **(B, n = 9391** **)**, other **(C, n = 4081** **)** and unknown febrile **(D, n = 5141** **)** infectious episode rates by season, divided into febrile (red line) and non-febrile (blue line).

The total number of infectious episodes was 71,578 with an average of 1.6 ICD-10 codes reported per episode. Out of the total infectious episodes 52,965 (74.0%) were respiratory infectious episodes, 9,391 (13.1%) were gastrointestinal infectious episodes, 4,081 (5.7%) were other infectious episodes and 5,141 (7.2%) were unknown febrile infections (Table 1). Seasonal patterns for infectious episode rates are shown in Figure 2. Respiratory and gastrointestinal infectious episode rates were more frequent during winter-season, whereas no seasonal trend was observed for other infectious episodes or unknown febrile infections.

**Figure 3:**
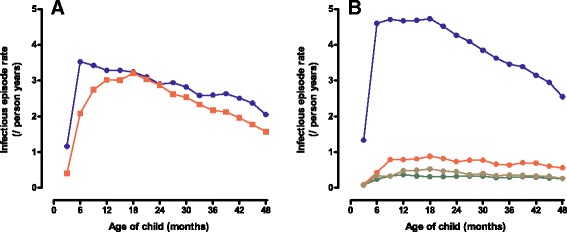
**Infectious episode rates by age.** Infectious episode rates by age is shown for febrile (red line) and non-febrile (blue line) in **A**, and for respiratory (blue line), gastrointestinal (red line), other (green line) and unknown febrile (brown line) in **B**.

Rates for infectious episodes by age are shown in Figure 3. The infectious episode rates peaked steeply at 6 months and started to decline after 18 months. The overall infectious episodes rates were 5.2 episodes per person year, including: respiratory infections 3.8 per person year, gastrointestinal infections 0.7 per person year, other known infection 0.3 per person year and unknown febrile infections 0.4 per person year. Peak rates for these infectious episodes were 4.7 for respiratory tract infections at 9 months, 1.0 for gastrointestinal infections at 18 months, 0.4 for other known infections at 12 months and 0.6 for unknown febrile infections at 18 months.

Incidence rate of infectious episodes was almost 50% higher in the European study sites as compared with US study sites (p < 0.001, Table 2). There was a slight excess of infectious episodes among males (rate ratio = 1.04, 95%CI = 1.02 – 1.05, p < 0.001). While the rate of infections differed significantly by the HLA-DR-DQ genotype (p < 0.001), the differences were small (Table 2). The FDR status was associated only with non-febrile infectious episodes (Table 2). When respiratory and gastrointestinal infectious episodes were evaluated separately, the associations with gender, FDR status and HLA-DR-DQ genotype were similar to that seen for all infectious episodes: However, within the US sites reporting of gastrointestinal infections was most frequent and respiratory infections less frequent in Colorado compared to Georgia/Florida and Washington State (data not shown).

**Table 2:** **Infectious episode rates by subject attributes**

**Factor**	**A. Any infectious episode rate ratios (RR)**			**B. Febrile infectious episode rate ratios (RR)**			**C. Non-febrile infectious episode rate ratios (RR)**		
	**RR**	**95%CI**	**p-value**	**RR**	**95%CI**	**p-value**	**RR**	**95%CI**	**p-value**
**Site**									
US-Colorado	1.00	ref		1.00	ref		1.00	ref	
US-Georgia	1.18	1.14 – 1.22		1.30	1.23 – 1.37		1.12	1.07 – 1.16	
US-Washington	1.05	1.02 – 1.09		1.03	0.97 – 1.09		1.06	1.02 – 1.10	
Europe – Finland	1.50	1.46 – 1.54		1.95	1.87 – 2.04		1.28	1.24 – 1.32	
Europe – Germany	1.40	1.34 – 1.45		1.80	1.69 – 1.92		1.19	1.13 – 1.25	
Europe - Sweden	1.42	1.39 – 1.46	<0.001	2.39	2.30 – 2.49	<0.001	0.94	0.91 – 0.97	<0.001
**Gender of child**									
Female	1.00	ref		1.00	ref		1.00	ref	
Male	1.04	1.02 – 1.05	<0.001	1.03	1.00 – 1.05	0.02	1.05	1.03 – 1.07	<0.001
**First degree relative with T1D**									
No	1.00	ref		1.00	ref		1.00	ref	
Yes	1.01	0.98 – 1.04	0.40	0.97	0.93 – 1.01	0.19	1.05	1.01 – 1.09	0.02
**HLA-DR-DQ genotype**									
DR4-DQ8/DR4-DQ8	1.00	ref		1.00	ref		1.00	ref	
DR3-DQ2/DR4-DQ8	1.02	1.00 – 1.04		0.99	0.96 – 1.02		1.05	1.02 – 1.08	
DR4-DQ8/DR8-DQ4	0.97	0.94 – 0.99		0.97	0.93 – 1.01		0.96	0.93 – 1.00	
DR3-DQ2/DR3-DQ2	0.99	0.97 – 1.01		0.92	0.89 – 0.95		1.05	1.02 – 1.09	
HLA-FDR specific^a^	0.98	0.93 – 1.03	<0.001	1.01	0.94 – 1.09	<0.001	0.95	0.89 – 1.02	<0.001

^a^FDR HLA-DR-DQ genotypes are DR4-DQ8 with either DR4-DQ4.2, DR13-DQ6.4, DR12-DQ5.1, DR4-DQ7, DR4-DQ2, DR9-DQ9 or genotype DR3-DQ2/DR9-DQ9.

Multiple Poisson regression models examining the infectious episode rates by site, gender, HLA-DR-DQ genotype and family history of a first degree relative with T1D. Rate ratios show risk of A) any type of infectious episode, B) febrile and C) non-febrile infectious episode rates relative to reference group.

## Discussion

TEDDY study seeks to evaluate multiple environmental factors to identify those associated with the autoimmune process leading to T1D. Some environmental exposures can be assessed by analyzing the biological samples taken from the study subjects, but TEDDY book parental reporting system is equally important, as it gives day-to-day information on a large variety of exposures: symptomatic infections, other medical conditions, medications, dietary factors and psychosocial factors. Recording of multiple environmental exposures enables not only analysis of multiple types of exposures separately, but also analysis on possible interactions between the exposures on the risk of T1D. Infectious disease data will be included into multiple analyses within TEDDY aiming at elucidating the environmental risk factors, or protective factors, and their possible interactions in T1D pathogenesis.

Data processing is of crucial importance, as it strongly influences the data analyses and results that follow. Processing of infectious disease reports is not straightforward, as the processed data should allow analysis of single infectious agents as well as broader categories of infections. Infectious disease data processing should also take into account the possibility of multiple reports during the course of a single infection. Therefore, there is a clear need for standardization of the procedures used to process the data regarding infectious diseases prior to its use in different kind of studies. Here we describe such procedures which have been developed in the TEDDY study and which are applicable also to other large scale clinical studies. We are not aware of equally detailed description of infectious disease report processing in previous literature.

Symptoms of the common childhood infections, i.e. respiratory and gastrointestinal infections, are often non-specific and the causative microbe can usually not be concluded based on clinical picture. Furthermore, the course of the common infections is often mild and self-limited, which is why identification of the causative microbe by laboratory analyses is not necessary in normal clinical practice. For these reasons, a majority of childhood infections do not receive a microbe-specific diagnosis, but a diagnosis based on anatomical site of infection and its symptoms. Also, in the present study, the majority of the infectious disease reports were not microbe-specific but based on the site of the infection and symptoms it caused (Additional file 5). These data are therefore not optimal for the identification of specific microbes causing these infections, unless the microbe of interest happens to have a distinct clinical presentation, which usually leads to correct microbe specific diagnosis, e.g., in varicella. In addition, causative microbes may become identified if they cause severe infections, which require efficient treatment and rigorous laboratory analyses, e.g., in meningitis or streptococcal infection.

One might criticize inclusion of gastrointestinal symptoms in the “Respiratory infectious episodes”. This decision was justified by the fact that nausea and vomiting in young children are not straightforwardly indicative of a gastrointestinal infection, as these symptoms can also be induced by many respiratory infections [5,6]. We therefore decided to interpret nausea and vomiting as symptoms of respiratory tract infection, if these symptoms occurred together with respiratory tract symptoms/diagnoses. This may possibly lead to underestimation of the number of gastrointestinal infections. Still, we feel that our interpretation is justified in the present study evaluating infections in these young 0–4 year old children. One might also ask why conjunctivitis is included into “Respiratory infectious episodes”. This inclusion was made as conjunctivitis in children is often caused by same viruses and bacteria causing upper respiratory infections, and in these cases both conjunctival and respiratory symptoms present usually simultaneously [6,7]. Enterovirus infections were also included into “Respiratory infectious episodes”, since symptoms of non-polio enteroviruses manifest most often in respiratory tract as e.g. herpangina, hand, foot and mouth syndrome, pharyngitis and croup [8]. Mild febrile illness with or without rash is also common, as well as conjunctivitis, whereas the more severe and less common manifestations include meningitis, encephalitis, myocarditis and sepsis [8]. Gastroenteral symptoms are rare, although enteroviruses replicate also in the intestinal lymphoid tissue and are often detectable in stool during infection.

The total number of ICD-10 code reports extracted from database was 113,884. If we had taken each of these as an indication of an infection, we would have had a mean of 8.2 annual infections per child. This would probably have been an overestimation of the number of symptomatic infections, as a single microbe often generates multiple symptoms and diagnoses as the infection spreads to different anatomical sites. Many of the extracted ICD-10 codes were reported within a week, many even on same day, and if these codes belonged to the same main group of codes (Table 1) they were merged into a single infectious episode. Each episode may thus include several diagnoses and/or symptoms, e.g. common cold together with middle ear infection and fever, but we assume that there is only one microbe causing these. By this ICD-10 code merging procedure we ended up with 71,578 infectious episodes, translating to a mean of 5.2 annual infectious episodes per child. This rate is comparable to the extrapolated yearly rate of infectious events in the BABYDIET study cohort during first three years of life [9]. Most of the infectious episodes were respiratory tract infections, as expected. The incidence of respiratory infectious episodes, the shape of the slope in the incidence according to age, as well as the higher incidence in winter period are all in line with previous reports on occurrence of respiratory infections in young children [10,11]. Also in gastrointestinal disease outbreaks a clear seasonal distribution has been observed with more cases during winter months [12], and the same observation was made in the present study. These findings suggest reasonable soundness and validity for the used data collection, processing and infectious episode approach.

Infectious episode rates were slightly higher in males as compared with females. Several studies have reported gender differences in the incidence and severity of infectious diseases. Higher incidence of most respiratory tract infections has been reported in males [13,14], which may account for the male preponderance in the present study, as most of the infectious episodes in this study were of respiratory type.

HLA has been shown to influence the severity and outcome of many viral and bacterial infections [15-18]. Slight differences between HLA groups were observed also in the present study. Having HLA-DR4-DQ8 seemed to increase the risk of febrile infections, whereas non-febrile infections were more common in the HLA-DR3-DQ2 group. When looking at febrile and non-febrile infections together, highest rate was seen in the HLA-DR-DQ 3-2/4-8 group. These differences between the HLA groups may be due to different infectious disease susceptibility due to differences in antigen presentation.

While reporting of infectious episodes was notably more frequent in European sites as compared with US sites, the rates were relatively similar within sites from each continent. There was a minor cross-continental difference in recording fever, as in Europe the threshold for fever was slightly lower (38°) than in the US (101°F equals to 38,33°C). Differences in access to child-care, exposure risk to infections, hyper vigilance among some families from countries with high incidence for T1D, and reporting bias of infections are some explanations for the differences in rates between sites. However, these explanations are unlikely to explain the statistically significant differences in infectious episode rates between HLA-DR-DQ groups as parents were not told of their child’s specific HLA risk group. Therefore the risk of exposure and reporting bias are likely to be non-differential between groups. Future work will examine demographic and psychosocial factors in relation to the reported infectious episode rates, and compare the reported infection rates with biomarkers determined from biological samples.

This study has its limitations. Parental reporting of infectious diseases captures only symptomatic infections, which is generally considered to represent only a fraction of all microbial exposures. Reporting of infections can also be prone to variation between families due to varying level of study compliance and vigilance in noticing symptoms. Another possible limitation is the pedantry of ICD-10 coding system, which can lead to variation in translation of parental reports into ICD-10 codes, especially if countries have different national guidelines and conventions in using ICD-10 codes. Possible variations in the use of ICD-10 codes can be overcome by using the infectious episode approach developed in this study, as in this mode of analysis same sort of infections fall under same category even if the infections would have been recorded using slightly different ICD-10 codes. We also noticed, that it was sometimes difficult for the research nurses to differentiate between ICD-10 codes for acute and chronic infections, since some ICD-10 codes for chronic infections had been entered into the database as acute illnesses. The total number of chronic infections misclassified as acute infections in the database was low (n = 116) by chronic middle ear infections accounting for most (n = 97, detailed data not shown).

## Conclusions

Several processing steps are required before parental reports of children's symptoms and illnesses are ready for analysis. In this paper we have described how parental data is processed into individual ICD-10 codes, infectious disease categories and infectious episodes in TEDDY study. These data levels enable time dependent analysis of single infectious agents as well as larger arrays of infectious agents or clinical disease entities, and they are suitable also for analyses of interactions between infections and other environmental exposures in T1D pathogenesis. The primary descriptive data of infections during TEDDY follow-up fits well with general knowledge of infectious disease epidemiology. This study protocol for infectious disease data processing can be used as a standard in the forthcoming analyses within TEDDY and also as a model to document infectious exposures in other epidemiological studies.

## Additional files

Additional file 1:
**The TEDDY Study Group.**
Additional file 2:
**The TEDDY book page for illness reports for 0–2 year old children.**
Additional file 3:
**The TEDDY book page for illness reports for 2–5 year old children.**
Additional file 4:
**Data extraction form for acute illnesses.**
Additional file 5:
**Categories of infections.**

## Abbreviations

CI: Confidence interval
HLA: Human leukocyte antigen
ICD: International classification of disease
T1D: Type 1 diabetes
TEDDY: The environmental determinants of diabetes in the young
