# Supplementary material for: A method for reporting and classifying acute infectious diseases in a prospective study of young children: TEDDY
Source: BMC Pediatr. 2015 Mar 20;15:24. doi: 10.1186/s12887-015-0333-8 (PMC4377063; doi:10.1186/s12887-015-0333-8)
Supplement: Additional file 1: — The TEDDY Study Group. [file 12887_2015_333_MOESM1_ESM.docx]

**Additiona file 1.**

**The Teddy Study Group**

**Colorado Clinical Center:** Marian Rewers, M.D., Ph.D., PI^1,4,5,6,10,11^, Katherine Barriga^12^, Kimberly Bautista^12^, Judith Baxter^9,12,15^, Ruth Bedoy^2^, George Eisenbarth, M.D., Ph.D., Nicole Frank^2^, Patricia Gesualdo^2,6,12,14,15^, Michelle Hoffman^12,13,14^, Lisa Ide, Rachel Karban^12^, Edwin Liu, M.D.^13^, Jill Norris, Ph.D.^2,3,12^, Kathleen Waugh^6,7,12,15^, Adela Samper-Imaz, Andrea Steck, M.D.^3,14^. University of Colorado, Anschutz Medical Campus, Barbara Davis Center for Childhood Diabetes.

**Georgia/Florida Clinical Center:** Jin-Xiong She, Ph.D., PI^1,3,4,11,^†, Desmond Schatz, M.D.*^4,5,7,8^, Diane Hopkins^12^, Leigh Steed^12,13,14,15^, Jamie Thomas*^6,12^, Katherine Silvis^2^, Michael Haller, M.D.*^14^, Meena Shankar*^2^, Eleni Sheehan*, Melissa Gardiner, Richard McIndoe, Ph.D., Haitao Liu, M.D.†, John Nechtman†, Ashok Sharma, Joshua Williams, Gabriela Foghis, Stephen W. Anderson, M.D.^^^. Medical College of Georgia, Georgia Regents University. *University of Florida, †Jinfiniti Biosciences LLC, Augusta, GA, ^^^Pediatric Endocrine Associates, Atlanta, GA.

**Germany Clinical Center:** Anette G. Ziegler, M.D., PI^1,3,4,11^, Andreas Beyerlein Ph.D.^2^, Ezio Bonifacio Ph.D.*^5^, Michael Hummel, M.D.^13^, Sandra Hummel, Ph.D.^2^, Kristina Foterek^¥2^, Mathilde Kersting, Ph.D.^¥2^, Annette Knopff^7^, Sibylle Koletzko, M.D.^¶13^, Claudia Peplow^12^, Roswith Roth, Ph.D.^9^, Julia Schenkel^2,12^, Joanna Stock^9,12^, Elisabeth Strauss^12^, Katharina Warncke, M.D.^14^, Christiane Winkler, Ph.D.^2,12,15^. Forschergruppe Diabetes e.V. at Helmholtz Zentrum München. *Center for Regenerative Therapies, TU Dresden, ^¶^Dr. von Hauner Children´s Hospital, Department of Gastroenterology, Ludwig Maximillians University Munich, ^¥^Research Institute for Child Nutrition, Dortmund.

**Finland Clinical Center:** Olli G. Simell, M.D., Ph.D., PI^¥^1,4,11,13^, Jorma Toppari, M.D., Ph.D., Co-PI^4^, Heikki Hyöty, M.D., Ph.D.*^±6^, Jorma Ilonen, M.D., Ph.D.^¥ ¶3^, Miia Kähönen^µ¤^, Mikael Knip, M.D., Ph.D.*^±5^, Annika Koivu^¥^^, Mirva Koreasalo*^±§2^, Kalle Kurppa, Maria Lönnrot, M.D., Ph.D.*^±6^, Elina Mäntymäki^¥^^, Katja Multasuo^µ¤^, Juha Mykkänen, Ph.D.^^¥ 3^, Kirsti Näntö-Salonen, M.D., Ph.D.^¥^12^, Tiina Niininen^±^*^12^, Mia Nyblom*^±^, Jenna Rautanen^±§^, Anne Riikonen*^±^, Minna Romo^¥^^, Aaro Simell^¥^^, Barbara Simell^¥^9,12,15^, Satu Simell, Tuula Simell, Ph.D.^¥^9,12^, Ville Simell^^¥13^, Maija Sjöberg^¥^12,14^, Aino Stenius^µ¤12^, Eeva Varjonen^¥^12^, Riitta Veijola, M.D., Ph.D.^µ¤14^, Suvi M. Virtanen, M.D., Ph.D.*^±§2^, Mari Åkerlund*^±§^. ^¥^University of Turku, *University of Tampere, ^µ^University of Oulu, ^^^Turku University Hospital, ^±^Tampere University Hospital, ^¤^Oulu University Hospital, §National Institute for Health and Welfare, Finland, ^¶^University of Kuopio.

**Sweden Clinical Center:** Åke Lernmark, Ph.D., PI^1,3,4,5,6,8,10,11,15^, Daniel Agardh, M.D., Ph.D.^13^, Carin Andrén-Aronsson^2,13^, Maria Ask, Jenny Bremer, Ulla-Marie Carlsson, Corrado Cilio, Ph.D., M.D.^5^, Emilie Ericson-Hallström^2^, Lina Fransson, Thomas Gard, Joanna Gerardsson, Rasmus Håkansson, Monica Hansen, Gertie Hansson^12,14^, Susanne Hyberg, Fredrik Johansen, Berglind Jonasdottir M.D., Linda Jonsson, Helena Larsson M.D., Ph.D. ^6,14^, Barbro Lernmark, Ph.D., Maria Månsson-Martinez, Maria Markan, Theodosia Massadakis, Jessica Melin^12^, Zeliha Mestan, Kobra Rahmati, Anita Ramelius, Anna Rosenquist, Monica Sedig Järvirova, Sara Sibthorpe, Birgitta Sjöberg, Ulrica Swartling, Ph.D.^9,12^, Erika Trulsson, Carina Törn, Ph.D. ^3,15^, Anne Wallin, Åsa Wimar^12^, Sofie Åberg. Lund University.

**Washington Clinical Center:** William A. Hagopian, M.D., Ph.D., PI^1,3,4, 5, 6,7,11,13, 14^, Xiang Yan, M.D., Michael Killian^6,7,12,13^, Claire Cowen Crouch^12,14,15^, Jennifer Skidmore^2^, Stephen Ayres, Kayleen Dunson, Diana Heaney, Rachel Hervey, Renee Kindschi, Arlene Meyer, Denise Mulenga, Elizabeth Scott, Joshua Stabbert, Nancy Williams, John Willis. Pacific Northwest Diabetes Research Institute.

**Pennsylvania Satellite Center:** Dorothy Becker, M.D., Margaret Franciscus^12^, MaryEllen Dalmagro-Elias Smith^2^, Ashi Daftary, M.D., Mary Beth Klein. Children’s Hospital of Pittsburgh of UPMC.

**Data Coordinating Center:** Jeffrey P. Krischer, Ph.D.,PI^1,4,5,10,11^, Michael Abbondondolo, Sarah Austin-Gonzalez, Rasheedah Brown^12,15^, Brant Burkhardt, Ph.D.^5,6^, Martha Butterworth^2^, David Cuthbertson, Christopher Eberhard, Steven Fiske^9^, Veena Gowda, David Hadley, Ph.D.^3,13^, Hye-Seung Lee, Ph.D.^1,2,13,15^, Shu Liu, Xiang Liu, Ph.D.^2,9,12^, Kristian Lynch, Ph.D. ^5,6,9,15^, Jamie Malloy, Cristina McCarthy^12,15^, Wendy McLeod^2,5,6,13,15^, Laura Smith, Ph.D.^9,12^, Susan Smith^12,15^, Roy Tamura, Ph.D.^1,2,13^, Ulla Uusitalo, Ph.D.^2,15^, Kendra Vehik, Ph.D.^4,5,6,14,15^, Earnest Washington, Jimin Yang, Ph.D., R.D.^2,15^. University of South Florida.

**Project scientist:** Beena Akolkar, Ph.D.^1,3,4,5, 6,7,10,11^. National Institutes of Diabetes and Digestive and Kidney Diseases.

**Other contributors:** Kasia Bourcier, Ph.D.^5^, National Institutes of Allergy and Infectious Diseases. Thomas Briese, Ph.D.^6,15^, Columbia University. Suzanne Bennett Johnson, Ph.D.^9,12^, Florida State University. Steve Oberste, Ph.D.^6^, Centers for Disease Control and Prevention. Eric Triplett, Ph.D.^6^, University of Florida.

**Autoantibody Reference Laboratories:** Liping Yu, M.D.^^ 5^, Dongmei Miao, M.D.^^^, Polly Bingley, M.D., FRCP*^5^, Alistair Williams*, Kyla Chandler*, Saba Rokni*, Joanna Boldison*, Jacob Butterly*, Jessica Broadhurst*, Gabriella Carreno*, Claire Caygill*, Rachel Curnock*, Peter Easton*, Ivey Geoghan*, Julia Goode*, Anna Long*, Molly Payne*, James Pearson*, Charles Reed*, Sophie Ridewood*, Rebecca Wyatt*. ^^^Barbara Davis Center for Childhood Diabetes, University of Colorado Denver, *School of Clinical Sciences, University of Bristol UK.

**Cortisol Laboratory:** Elisabeth Aardal Eriksson, M.D., Ph.D., Ewa Lönn Karlsson. Department of Clinical Chemistry, Linköping University Hospital, Linköping, Sweden.

**Dietary Biomarkers Laboratory:** Iris Erlund, Ph.D.^2^, Irma Salminen, Jouko Sundvall, Jaana Leiviskä, Mari Lehtonen, Ph.D. National Institute for Health and Welfare, Helsinki, Finland.

**HbA1c Laboratory:** Randie R. Little, Ph.D., Alethea L. Tennill. Diabetes Diagnostic Laboratory, Dept. of Pathology, University of Missouri School of Medicine.

**HLA Reference Laboratory:** Henry Erlich, Ph.D.^3^, Steven J. Mack, Ph.D., Anna Lisa Fear. Center for Genetics, Children’s Hospital Oakland Research Institute.

**Metabolomics Laboratory:** Oliver Fiehn, Ph.D., Bill Wikoff, Ph.D., Tobias Kind, Ph.D., Mine Palazoglu, Joyce Wong, Gert Wohlgemuth. UC Davis Metabolomics Center.

**Microbiome and Viral Metagenomics Laboratory:** Joseph F. Petrosino, Ph.D.^6^. Alkek Center for Metagenomics and Microbiome Research, Department of Molecular Virology and Microbiology, Baylor College of Medicine.

**OGTT Laboratory:** Santica M. Marcovina, Ph.D., Sc.D., Vinod P. Gaur, Ph.D., Northwest Lipid Metabolism and Diabetes Research Laboratories, University of Washington.

**Repository:** Heather Higgins, Sandra Ke. NIDDK Biosample Repository at Fisher BioServices.

**RNA Laboratory and Gene Expression Laboratory:** Jin-Xiong She, Ph.D., PI^1,3,4,11^, Richard McIndoe, Ph.D., Haitao Liu, M.D., John Nechtman, Yansheng Zhao, Na Jiang, M.D. Jinfiniti Biosciences, LLC.

**SNP Laboratory:** Stephen S. Rich, Ph.D.^3^, Wei-Min Chen, Ph.D.^3^, Suna Onengut-Gumuscu, Ph.D.^3^, Emily Farber, Rebecca Roche Pickin, Ph.D., Jordan Davis, Dan Gallo. Center for Public Health Genomics, University of Virginia.

***Committees:***

^1^Ancillary Studies, ^2^Diet, ^3^Genetics, ^4^Human Subjects/Publicity/Publications, ^5^Immune Markers, ^6^Infectious Agents, ^7^Laboratory Implementation, ^8^Maternal Studies, ^9^Psychosocial, ^10^Quality Assurance, ^11^Steering, ^12^Study Coordinators, ^13^Celiac Disease, ^14^Clinical Implementation, ^15^Quality Assurance Subcommittee on Data Quality.
