# Supplementary material for: A method for reporting and classifying acute infectious diseases in a prospective study of young children: TEDDY
Source: BMC Pediatr. 2015 Mar 20;15:24. doi: 10.1186/s12887-015-0333-8 (PMC4377063; doi:10.1186/s12887-015-0333-8)
Supplement: Additional file 2: — The TEDDY book page for illness reports for 0–2 year old children. [file 12887_2015_333_MOESM2_ESM.pdf]

ILLNESSES

We want to know if your child has been ill. Below is a list of illnesses/symptoms that sick children sometimes have.

- **Diarrhea**—four or more loose or watery stools a day, more loose and watery than normal for your child.
- **Vomiting**—not just usual "spitting up" after meals.
- **Rash**—red, blotchy skin, or blisters. Please note whether the rash is raised or flat as well as its location on the body (e.g., cheeks, trunk, fingers).
- **Skin infections**—impetigo, boils (not eczema).
- **Poor appetite and/or very fussy**—lasting at least 24 hours.
- **Any illness with fever**—temperature above 38°C/101° F.
- **Pink eye or other eye infection**
- **Ear infection or ear pain**
- **Short of breath**—for at least 1 full day with rapid breathing, wheezing, or difficult breathing.
- **Sore throat**
- **Strep throat**—diagnosed by throat culture.
- **Headache**
- **Cold, runny nose and/or cough**
- **Bronchitis**
- **Pneumonia**
- **Croup or RSV (Respiratory Syncytial Virus)**
- **Influenza**—high fever associated with general malaise and muscle pain, occurring during an influenza season (not including stomach flu).
- **Mouth sores**—cold sores, ulcers.
- **Parasites**—pinworms, giardia, etc.
- **Jaundice**—yellow color of gums and white part of eyes.
- **Seizure**—due to fever.
- **Seizure**—due to other causes.
- **Urinary tract infection**
- **Any illness requiring help (phone or visit) from doctor or nurse**
- **Yeast infection**
- **Other**

Please record every time your child has been sick. Write as many symptoms as necessary. If your child is ill with symptoms not listed above, please write these in the table as well.

Date illness first appeared (DD/MMM/YYYY)	Illness/symptoms	Fever? (temperature is equal to or higher than 38°C or 101°F)	Did you take your baby to a health care provider for this illness?
		[ ] No [ ] Yes	[ ] No [ ] Yes
		[ ] No [ ] Yes	[ ] No [ ] Yes
		[ ] No [ ] Yes	[ ] No [ ] Yes
		[ ] No [ ] Yes	[ ] No [ ] Yes
		[ ] No [ ] Yes	[ ] No [ ] Yes
		[ ] No [ ] Yes	[ ] No [ ] Yes
		[ ] No [ ] Yes	[ ] No [ ] Yes
		[ ] No [ ] Yes	[ ] No [ ] Yes
		[ ] No [ ] Yes	[ ] No [ ] Yes
		[ ] No [ ] Yes	[ ] No [ ] Yes
		[ ] No [ ] Yes	[ ] No [ ] Yes
