# Supplementary material for: A method for reporting and classifying acute infectious diseases in a prospective study of young children: TEDDY
Source: BMC Pediatr. 2015 Mar 20;15:24. doi: 10.1186/s12887-015-0333-8 (PMC4377063; doi:10.1186/s12887-015-0333-8)
Supplement: Additional file 3: — The TEDDY book page for illness reports for 2–5 year old children. [file 12887_2015_333_MOESM3_ESM.pdf]

ILLNESSES

We want to know if your child has been ill. Below is a list of illnesses/symptoms that sick children sometimes have.

- **Diarrhea** - four or more loose or watery stools a day, more loose and watery than normal for your child.
- **Vomiting** - not just usual "spitting up" after meals.
- **Rash** - red, blotchy skin, or blisters. Please note whether the rash is raised or flat as well as its location on the body (ex: cheeks, trunk, fingers).
- **Skin infections** - impetigo, boils (not eczema).
- **Poor appetite and/or very fussy** - lasting at least 24 hours.
- **Any illness with fever** - temperature above 38°C/101° F.
- **Pink eye or other eye infection**
- **Ear infection or ear pain**
- **Short of breath** - for at least 1 full day with rapid breathing, wheezing, or difficult breathing.
- **Sore throat**
- **Strep throat** - diagnosed by throat culture.
- **Headache**
- **Cold, runny nose and/or cough**
- **Bronchitis**
- **Pneumonia**
- **Croup or RSV (Respiratory Syncytial Virus)**
- **Influenza** - high fever associated with general malaise and muscle pain, occurring during an influenza season (**not** including stomach flu).
- **Mouth sores** - cold sores, ulcers.
- **Parasites** - pinworms, giardia, etc.
- **Jaundice** - yellow color of gums and white part of eyes.
- **Seizure** - due to fever.
- **Seizure** - due to other causes.
- **Urinary tract infection**
- **Any illness requiring help (phone or visit) from doctor or nurse.**
- **Yeast infection**
- **Other**

Please record <u>every time your child has been sick</u>. Write as many symptoms as necessary. If your child is ill with symptoms not listed above, please write these in the table as well.

Date illness first appeared DD/MMM/YYYY	Symptoms	Fever? (temperature is equal to or higher than 38°C or 101°F)	Diagnosis
		[ ] No<br>[ ] Yes, measured<br>[ ] Yes, not measured	______________<br>[ ] Diagnosed by you<br>[ ] Diagnosed by health care provider
		[ ] No<br>[ ] Yes, measured<br>[ ] Yes, not measured	______________<br>[ ] Diagnosed by you<br>[ ] Diagnosed by health care provider
		[ ] No<br>[ ] Yes, measured<br>[ ] Yes, not measured	______________<br>[ ] Diagnosed by you<br>[ ] Diagnosed by health care provider
		[ ] No<br>[ ] Yes, measured<br>[ ] Yes, not measured	______________<br>[ ] Diagnosed by you<br>[ ] Diagnosed by health care provider
