# Supplementary material for: A method for reporting and classifying acute infectious diseases in a prospective study of young children: TEDDY
Source: BMC Pediatr. 2015 Mar 20;15:24. doi: 10.1186/s12887-015-0333-8 (PMC4377063; doi:10.1186/s12887-015-0333-8)
Supplement: Additional file 4: — Data extraction form for acute illnesses. [file 12887_2015_333_MOESM4_ESM.pdf]

7a Acute Illnesses (question on pages 14-16) -*Has the child been ill since the last visit? Record all chronic illnesses/conditions on the next page.*

○ No (Continue to the next section) ○ Yes (fill in the table)

Local Use Only [ ]

SubjectID [ ]

Date illness first appeared (DD/MMM/YYYY)	ICD-10 Code: ONLY code Symptoms here - (ALWAYS CODE SYMPTOMS)	Fever? (temperature is equal to or higher than 38°C or 101°F)	Diagnosis	
__ / ___ / ____ Symptoms ____	___ . _ ___ . _ ___ . _ ○ No symptoms	○ No ○ Yes, Measured ○ Yes, Not Measured	____ Diagnosis	ICD-10 Code ___ . _ ○ Diagnosed by parent ○ Diagnosed by health care provider
__ / ___ / ____ Symptoms ____	___ . _ ___ . _ ___ . _ ○ No symptoms	○ No ○ Yes, Measured ○ Yes, Not Measured	____ Diagnosis	ICD-10 Code ___ . _ ○ Diagnosed by parent ○ Diagnosed by health care provider
__ / ___ / ____ Symptoms ____	___ . _ ___ . _ ___ . _ ○ No symptoms	○ No ○ Yes, Measured ○ Yes, Not Measured	____ Diagnosis	ICD-10 Code ___ . _ ○ Diagnosed by parent ○ Diagnosed by health care provider
__ / ___ / ____ Symptoms ____	___ . _ ___ . _ ___ . _ ○ No symptoms	○ No ○ Yes, Measured ○ Yes, Not Measured	____ Diagnosis	ICD-10 Code ___ . _ ○ Diagnosed by parent ○ Diagnosed by health care provider
__ / ___ / ____ Symptoms ____	___ . _ ___ . _ ___ . _ ○ No symptoms	○ No ○ Yes, Measured ○ Yes, Not Measured	____ Diagnosis	ICD-10 Code ___ . _ ○ Diagnosed by parent ○ Diagnosed by health care provider

37603
