# Supplementary material for: A method for reporting and classifying acute infectious diseases in a prospective study of young children: TEDDY
Source: BMC Pediatr. 2015 Mar 20;15:24. doi: 10.1186/s12887-015-0333-8 (PMC4377063; doi:10.1186/s12887-015-0333-8)
Supplement: Additional file 5: — Categories of infections. [file 12887_2015_333_MOESM5_ESM.docx]

**Additional file 5. Categories of infections**

Categories of infections formed by grouping all reported ICD-10 codes for infectious diseases and fever reports into clinically relevant entities. Altogether 113,884 ICD-10 codes for infections and 54,501"yes"-fever reports were categorized. Every ICD-10 code with three characters comprises also all its sub-codes with four characters, unless otherwise indicated.

|  |  | **Diagnosis by** | | | **total** | **% of**  **ICD-10 codes** |
| --- | --- | --- | --- | --- | --- | --- |
|  |  | **Health**  **Care**  **Provider**  **n** | **Parent**  **n** | **No**  **Data**  **n** | **n** |  |
| **1.** | **FEVER** |  |  |  |  |  |
|  |  |  |  |  |  |  |
| **1.1.** | **Yes-answer in TEDDY book question on fever** | **25828** | **20984** | **7989** | **54501** |  |
|  | ICD-10 code reported with yes-answer |  |  |  | 54216 |  |
|  | No ICD-10 code reported with yes-answer |  |  |  | 285 |  |
|  |  |  |  |  |  |  |
| **1.2.** | **ICD-10 codes for fever** | **3840** | **7501** | **2273** | **13614** | **12.0** |
| R50 | Fever of other and unknown origin |  |  |  | 13614 |  |
|  |  |  |  |  |  |  |
| **2.** | **VIRUS INFECTIONS,**  **not elsewhere classified** | **16804** | **25583** | **11032** | **53419** |  |
|  |  |  |  |  |  |  |
| **2.1.** | **Common cold** | **14059** | **23530** | **10735** | **48324** | **42.4** |
| J00 | **Acute nasopharyngitis [common cold]** |  |  |  | 11935 |  |
| J02 | Acute pharyngitis **(excluding J02.0 Streptococcal pharyngitis)** |  |  |  | 1145 |  |
| **J06** | Acute upper respiratory infections of multiple and unspecified sites |  |  |  | 35194 |  |
| **J31** | Chronic rhinitis, nasopharyngitis and pharyngitis |  |  |  | 1 |  |
| R06.7 | Sneezing |  |  |  | 49 |  |
|  |  |  |  |  |  |  |
| **2.2.** | **Laryngitis and tracheitis** | **853** | **242** | **53** | **1148** | **1.0** |
| J04 | Acute laryngitis and tracheitis |  |  |  | 198 |  |
| J05 | Acute obstructive laryngitis [croup] and epiglottitis |  |  |  | 950 |  |
| J37 | Chronic laryngitis and laryngotracheitis |  |  |  | 0 |  |
|  |  |  |  |  |  |  |
| **2.3.** | **Influenza** | **312** | **287** | **22** | **621** | **0.5** |
| J09 | Influenza due to certain identified influenza virus |  |  |  | 8 |  |
| J10 | Influenza due to other identified influenza virus |  |  |  | 25 |  |
| J11 | Influenza, virus not identified |  |  |  | 588 |  |
|  |  |  |  |  |  |  |
| **2.4.** | **Enterovirus** | **281** | **164** | **34** | **479** | **0.4** |
| B08.4 | Enteroviral vesicular stomatitis with exanthem |  |  |  | 444 |  |
| B08.5 | Enteroviral vesicular pharyngitis |  |  |  | 28 |  |
| B08.8 | Other specified viral infections characterized by skin and mucous membrane lesions |  |  |  | 4 |  |
| B34.1 | Enterovirus infection, unspecified site |  |  |  | 3 |  |
| B97.1 | Enterovirus as the cause of diseases classified to other chapters |  |  |  | 0 |  |
|  |  |  |  |  |  |  |
| **2.5.** | **Chicken pox/Varicella** | **161** | **770** | **109** | **1040** | **0.9** |
| B01 | [**Varicella [chickenpox]**](http://www.who.int/classifications/apps/icd/icd10online/gB00.htm#B01) |  |  |  | 1040 |  |
|  |  |  |  |  |  |  |
| **2.6.** | **Zoster** | **13** | **4** | **1** | **18** | **<0.1** |
| B02 | Zoster [herpes zoster] |  |  |  | 18 |  |
|  |  |  |  |  |  |  |
| **2.7.** | **Erythema infectiosum [fifth disease, parvovirus]** | **40** | **19** | **2** | **61** | **<0.1** |
| B08.3 | Erythema infectiosum [fifth disease] |  |  |  | 61 |  |
|  |  |  |  |  |  |  |
| **2.8.** | **Exanthema subitum** | **219** | **273** | **16** | **508** | **0.4** |
| B08.2 | Exanthema subitum [sixth disease] |  |  |  | 508 |  |
|  |  |  |  |  |  |  |
| **2.9.** | **Measles** | **0** | **0** | **0** | **0** | **0** |
| B05 | Measles |  |  |  | 0 |  |
|  |  |  |  |  |  |  |
| **2.10** | **Mumps** | **0** | **0** | **0** | **0** | **0** |
| B26 | Mumps |  |  |  | 0 |  |
|  |  |  |  |  |  |  |
| **2.11.** | **Rubella** | **0** | **2** | **0** | **2** | **<0.1** |
| B06 | Rubella [German measles] |  |  |  | 2 |  |
|  |  |  |  |  |  |  |
| **2.12.** | **Herpes simplex virus infection** | **90** | **49** | **11** | **150** | **0.1** |
| A60 | Anogenital herpesviral infection |  |  |  | 0 |  |
| B00 | Herpesviral [herpes simplex] infections |  |  |  | 150 |  |
|  |  |  |  |  |  |  |
| **2.13.** | **Other viral rash** | **28** | **10** | **3** | **41** | **<0.1** |
| B08 | **(the three-character code only) Other viral infections characterized by skin and mucous membrane lesions, not elsewhere classified** |  |  |  | 13 |  |
| B09 | **Unspecified viral infection characterized by skin and mucous membrane lesions** |  |  |  | **28** |  |
|  |  |  |  |  |  |  |
| **2.14.** | **Infectious mononucleosis** | **6** | **0** | **0** | **6** | **<0.1** |
| B27 | Infectious mononuclesosis |  |  |  | 6 |  |
|  |  |  |  |  |  |  |
| **2.15.** | **Respiratory syncytial virus infection** | **133** | **4** | **1** | **138** | **0.1** |
| J12.1 | Respiratory syncytial virus pneumonia |  |  |  | 23 |  |
| J20.5 | Acute bronchitis due to respiratory syncytial virus |  |  |  | 2 |  |
| J21.0 | Acute bronchiolitis due to respiratory syncytial virus |  |  |  | 10 |  |
| B97.4 | Respiratory syncytial virus as the cause of diseases classified to other chapters |  |  |  | 103 |  |
|  |  |  |  |  |  |  |
| **2.16.** | **Viral infections of the central nervous system, not elsewhere classified** | **0** | **0** | **0** | **0** | **0** |
| A80 | Acute poliomyelitis |  |  |  | 0 |  |
| A81 | Atypical virus infections of central nervous system |  |  |  | 0 |  |
| A82 | Rabies |  |  |  | 0 |  |
| A83 | Mosquito-borne viral encephalitis |  |  |  | 0 |  |
| A84 | Tick-borne encephalitis |  |  |  | 0 |  |
| A85 | Other viral encephalitis, not elsewhere classified |  |  |  | 0 |  |
| A86 | Unspecified viral encephalitis |  |  |  | 0 |  |
| A87 | Viral meningitis |  |  |  | 0 |  |
| A88 | Other viral infections of central nervous system, not elsewhere classified |  |  |  | 0 |  |
| A89 | Unspecified viral infection of central nervous system |  |  |  | 0 |  |
|  |  |  |  |  |  |  |
| **2.17.** | **Other virus infections, not elsewhere classified** | **542** | **191** | **10** | **743** | **0.7** |
| A90 | Dengue fever [classical dengue] |  |  |  | 0 |  |
| A91 | Dengue haemorrhagic fever |  |  |  | 0 |  |
| A92 | Other mosquito-borne viral fevers |  |  |  | 0 |  |
| A93 | Other arhropod-borne viral fevers, not elsewhere classified |  |  |  | 3 |  |
| A94 | Unspecified arthropod-borne viral fever |  |  |  | 0 |  |
| A95 | Yellow fever |  |  |  | 0 |  |
| A96 | Arenaviral haemorrhagic fever |  |  |  | 0 |  |
| A98 | Other viral haemorrhagic fevers, not elsewhere classified |  |  |  | 1 |  |
| A99 | Unspecific viral haemorrhagic fever |  |  |  | 0 |  |
| B04 | Monkeypox |  |  |  | 0 |  |
| B08.0 | Other orthopoxvirus infections |  |  |  | 1 |  |
| B15 | Acute hepatitis A |  |  |  | 0 |  |
| B16 | Acute hepatitis B |  |  |  | 0 |  |
| B17 | Other acute viral hepatitis |  |  |  | 0 |  |
| B18 | Chronic viral hepatitis |  |  |  | 0 |  |
| B19 | Unspecified viral hepatitis |  |  |  | 0 |  |
| B24 | Unspecified human immunodeficiency virus (HIV) disease |  |  |  | 0 |  |
| B25 | Cytomegaloviral disease |  |  |  | 0 |  |
| B33 | Other viral diseases, not elsewhere classified |  |  |  | 1 |  |
| B34 | Viral infection of unspecific site **(excluding B34.1 Enterovirus infection, unspecified site)** |  |  |  | 735 |  |
| B97 | Viral agents as the cause of diseases classified to other chapters **(excluding B97.1 Enterovirus as the cause of diseases classified to other chapters and B97.4 Respiratory syncytial virus as the cause of diseases classified to other chapters)** |  |  |  | 2 |  |
|  |  |  |  |  |  |  |
| **2.18.** | **Viral warts and molluscum** | **67** | **38** | **35** | **140** | **0.1** |
| B07 | Viral warts |  |  |  | 44 |  |
| B08.1 | Molluscum contagiosum |  |  |  | 96 |  |
|  |  |  |  |  |  |  |
| **3.** | **TONSILLITIS OR STREPTOCOCCAL PHARYNGITIS** | **931** | **35** | **95** | **1061** | **0.9** |
|  |  |  |  |  |  |  |
| J02.0 | Streptococcal pharyngitis |  |  |  | 550 |  |
| J03 | Acute tonsillitis |  |  |  | 313 |  |
| J35.0 | Chronic tonsillitis |  |  |  | 0 |  |
| J36 | Peritonsillar abscess |  |  |  | 0 |  |
| R07.0 | Pain in throat |  |  |  | 198 |  |
|  |  |  |  |  |  |  |
| **4.** | **SINUSITIS** | **387** | **14** | **17** | **418** | **0.4** |
|  |  |  |  |  |  |  |
| J01 | Acute sinusitis |  |  |  | 416 |  |
| J32 | Chronic sinusitis |  |  |  | 2 |  |
|  |  |  |  |  |  |  |
| **5.** | **INFECTIONS OF EAR AND MASTOID PROCESS** | **11842** | **364** | **300** | **12506** | **11.0** |
|  |  |  |  |  |  |  |
| H65 | Nonsuppurative otitis media |  |  |  | 80 |  |
| H66 | Suppurative and unspecified otitis media |  |  |  | 9008 |  |
| H70 | Mastoiditis and related conditions |  |  |  | 2 |  |
| H73.0 | Acute myringitis |  |  |  | 2 |  |
| H73.1 | Chronic myringitis |  |  |  | 0 |  |
| H92 | Otalgia and effusion of ear |  |  |  | 3414 |  |
|  |  |  |  |  |  |  |
| **6.** | **BRONCHITIS AND LOWER RESPIRATORY INFECTIONS** | **6224** | **2525** | **4001** | **12750** |  |
|  |  |  |  |  |  |  |
| **6.1.** | **Symptoms** | **4835** | **2479** | **3966** | **11280** | **9.9** |
| R05 | Cough |  |  |  | 10478 |  |
| R06.2 | Wheezing |  |  |  | 802 |  |
|  |  |  |  |  |  |  |
| **6.2.** | **Bronchitis** | **696** | **33** | **20** | **749** | **0.7** |
| J20 | Acute bronchitis **(excluding J20.5 Acute bronchitis due to respiratory syncytial virus)** |  |  |  | 747 |  |
| J41 | Simple and mucopurulent chronic bronchitis |  |  |  | 0 |  |
| J42 | Unspecified chronic bronchitis |  |  |  | 2 |  |
|  |  |  |  |  |  |  |
| **6.3.** | **Bronchiolitis** | **233** | **8** | **9** | **250** | **0.2** |
| J21 | Acute bronchiolitis **(excluding J21.0 Acute bronchiolitis due to respiratory syncytial virus)** |  |  |  | 250 |  |
|  |  |  |  |  |  |  |
| **6.4.** | **Pneumonia** | **446** | **5** | **6** | **457** | **0.4** |
| J12 | Viral pneumonia, not elsewhere classified **(excluding J12.1 Respiratory syncytial virus pneumonia)** |  |  |  | 2 |  |
| J13 | Pneumonia due to Streptococcus pneumoniae |  |  |  | 1 |  |
| J14 | Pneumonia due to Haemophilus influenzae |  |  |  | 1 |  |
| J15 | Bacterial pneumonia, not elsewhere classified |  |  |  | 7 |  |
| J16 | Pneumonia due to other infectious organisms, not elsewhere classified |  |  |  | 0 |  |
| J18 | Pneumonia, organism unspecified |  |  |  | 446 |  |
|  |  |  |  |  |  |  |
| **6.5.** | **Other lower respiratory infection** | **14** | **0** | **0** | **14** | **<0.1** |
| J22 | Unspecified acute lower respiratory infection |  |  |  | 5 |  |
| J40 | Bronchitis, not specified as acute or chronic |  |  |  | 6 |  |
| J44.0 | Chronic obstructive pulmonary disease with acute lower respiratory infection |  |  |  | 0 |  |
| J85 | Abscess of lung and mediastinum |  |  |  | 1 |  |
| J86 | Pyothorax |  |  |  | 0 |  |
|  |  |  |  |  |  |  |
| **7.** | **INFECTIVE GASTROENTERITIS** | **1657** | **4389** | **1570** | **7616** |  |
|  |  |  |  |  |  |  |
| **7.1.** | **Bacterial gastroenteritis** | **22** | **16** | **5** | **43** | **<0.1** |
| A00 | Cholera |  |  |  | 0 |  |
| A01 | Typhoid and paratyphoid fevers |  |  |  | 0 |  |
| A02 | Other salmonella infections |  |  |  | 4 |  |
| A03 | Shigellosis |  |  |  | 0 |  |
| A04 | Other bacterial intestinal infections |  |  |  | 8 |  |
| A05 | Other bacterial foodborne infections, not elsewhere classified |  |  |  | 31 |  |
| **7.2.** | **Viral gastroenteritis** | **454** | **1312** | **24** | **1790** | **1.6** |
| A08 | Viral and other specified intestinal infections |  |  |  | 1790 |  |
| **7.3.** | **Other gastroenteritis** | **1181** | **3061** | **1541** | **5783** | **5.1** |
| A09 | Other gastroenteritis and colitis of infectious and unspecified origin |  |  |  | 5783 |  |
|  |  |  |  |  |  |  |
| **8.** | **GASTROENTERITIS SYMPTOMS** | **1456** | **4538** | **2350** | **8344** | **7.3** |
|  |  |  |  |  |  |  |
| R11 | Nausea and vomiting | 1456 | 4538 | 2350 | 8344 |  |
|  |  |  |  |  |  |  |
| **9.** | **PARASITES** | **35** | **94** | **27** | **156** | **0.1** |
|  |  |  |  |  |  |  |
| A06 | Amoebiasis |  |  |  | 4 |  |
| A07 | Other protozoal intestinal diseases |  |  |  | 13 |  |
| B50 | Plasmodium falciparum malaria |  |  |  | 1 |  |
| B51 | Plasmodium vivax malaria |  |  |  | 0 |  |
| B52 | Plasmodium malariae malaria |  |  |  | 0 |  |
| B53 | Other parasitologically confirmed malaria |  |  |  | 0 |  |
| B54 | Unspecified malaria |  |  |  | 0 |  |
| B55 | Leishmaniasis |  |  |  | 0 |  |
| B56 | African trypanosomiasis |  |  |  | 0 |  |
| B57 | Chagas disease |  |  |  | 0 |  |
| B58 | Toxoplasmosis |  |  |  | 0 |  |
| A59 | Trichomoniasis |  |  |  | 0 |  |
| B60 | Other protozpal diaseases, not elsewhere classified |  |  |  | 0 |  |
| B64 | Unspecified protozoal disease |  |  |  | 0 |  |
| B65 | Schistosomiasis [bilharziasis] |  |  |  | 0 |  |
| B66 | Other fluke infections |  |  |  | 0 |  |
| B67 | Echinococcosis |  |  |  | 0 |  |
| B68 | Taeniasis |  |  |  | 1 |  |
| B69 | Cysticercosis |  |  |  | 0 |  |
| B70 | Diphyllobothriasis and sparganosis |  |  |  | 0 |  |
| B71 | Other cestode infections |  |  |  | 0 |  |
| B72 | Dracunculiasis |  |  |  | 0 |  |
| B73 | Onchocerciasis |  |  |  | 0 |  |
| B74 | Filariasis |  |  |  | 0 |  |
| B75 | Trichinellosis |  |  |  | 0 |  |
| B76 | Hookworm diseases |  |  |  | 0 |  |
| B77 | Ascariasis |  |  |  | 0 |  |
| B78 | Strongyloidiasis |  |  |  | 0 |  |
| B79 | Trichuriasis |  |  |  | 0 |  |
| B80 | Enterobiasis |  |  |  | 121 |  |
| B81 | Other intestinal helminthiases, not elsewhere classified |  |  |  | 0 |  |
| B82 | Unspecified intestinal parasitism |  |  |  | 0 |  |
| B83 | Other helminthiases |  |  |  | 0 |  |
| B85 | Pediculosis and phthiriasis |  |  |  | 5 |  |
| B86 | Scabies |  |  |  | 9 |  |
| B87 | Myiasis |  |  |  | 0 |  |
| B88 | Other infestations |  |  |  | 1 |  |
| B89 | Unspecified parasitic disease |  |  |  | 1 |  |
|  |  |  |  |  |  |  |
| **10.** | **MYCOSES** | **198** | **54** | **14** | **266** | **0.2** |
|  |  |  |  |  |  |  |
| **B35** | **Dermatophytosis** |  |  |  | **60** |  |
| **B36** | **Other superficial mycoses** |  |  |  | **3** |  |
| **B37** | **Candidiasis** |  |  |  | **194** |  |
| **B38** | **Coccidioidomycosis** |  |  |  | **0** |  |
| **B39** | **Histoplasmosis** |  |  |  | **0** |  |
| **B40** | **Blastomycosis** |  |  |  | **0** |  |
| **B41** | **Paracoccidioidomycosis** |  |  |  | **0** |  |
| **B42** | **Sporotrichosis** |  |  |  | **0** |  |
| **B43** | **Chromomycosis and phaeomycotic abscess** |  |  |  | **0** |  |
| **B44** | **Aspergillosis** |  |  |  | **0** |  |
| **B45** | **Cryptococcosis** |  |  |  | **0** |  |
| **B46** | **Zygomycosis** |  |  |  | **0** |  |
| **B47** | **Mycetoma** |  |  |  | **0** |  |
| **B48** | **Other mycoses, not elsewhere classified** |  |  |  | **0** |  |
| **B49** | **Unspecified mycosis** |  |  |  | **9** |  |
|  |  |  |  |  |  |  |
| **11.** | **OTHER INFECTIONS** | **2784** | **777** | **173** | **3734** |  |
|  |  |  |  |  |  |  |
| **11.1.** | **Conjuctivitis** | **1761** | **699** | **129** | **2589** | **2.2** |
| B30 | Viral conjunctivitis |  |  |  | 7 |  |
| H10 | Conjunctivitis |  |  |  | 2582 |  |
|  |  |  |  |  |  |  |
| **11.2** | **Infections of external ear** | **16** | **0** | **0** | **16** | **<0.1** |
| H60 | Otitis externa **(excluding the non-infective codes H60.4 and H60.5** |  |  |  | 16 |  |
|  |  |  |  |  |  |  |
| **11.3.** | **Urinary tract infections** | **247** | **2** | **2** | **251** | **0.2** |
| N10 | Acute tubulo-interstitial nephritis |  |  |  | 15 |  |
| N11 | Chronic tubulo-interstitial nephritis |  |  |  | 2 |  |
| N12 | Tubulo-interstitial nephritis, not classified as acute or chronic |  |  |  | 0 |  |
| N30 | Cystitis |  |  |  | 11 |  |
| N39.0 | Urinary tract infection, site not specified |  |  |  | 223 |  |
|  |  |  |  |  |  |  |
| **11.4.** | **Other genitourinary infections** | **71** | **4** | **5** | **80** | **0.1** |
| N41.2 | Abscess of prostate |  |  |  | 0 |  |
| N45 | Orchitis and epididymitis |  |  |  | 1 |  |
| N48.1 | Balanoposthitis |  |  |  | 59 |  |
| N48.2 | Other inflammatory disorders of penis |  |  |  | 8 |  |
| N49 | Inflammatory disorders of male genital organs, not elsewhere classified |  |  |  | 1 |  |
| N70 | Salpingitis and oophoritis |  |  |  | 0 |  |
| N71 | Inflammatory disease of uterus, except cervix |  |  |  | 0 |  |
| N72 | Inflammatory disease of cervix uteri |  |  |  | 0 |  |
| N73 | Other female pelvic inflammatory diseases |  |  |  | 0 |  |
| N75 | Diseases of Bartholin gland |  |  |  | 0 |  |
| N76 | Other inflammation of vagina and vulva |  |  |  | 11 |  |
|  |  |  |  |  |  |  |
| **11.5.** | **Bacterial skin diseases** | **413** | **58** | **20** | **491** | **0.4** |
| A46 | Erysipelas |  |  |  | 0 |  |
| L00 | Staphylococcal scalded skin syndrome |  |  |  | 0 |  |
| L01 | Impetigo |  |  |  | 285 |  |
| L02 | Cutaneous abscess, furuncle and carbuncle |  |  |  | 48 |  |
| L03 | Cellulitis |  |  |  | 145 |  |
| L08 | Other local infections of skin and subcutaneous tissue |  |  |  | 13 |  |
|  |  |  |  |  |  |  |
| **11.6a.** | **Other bacterial diseases, not elsewhere classified (non-respiratory tract)** | **114** | **2** | **7** | **123** | **0.1** |
| A18 | Tuberculosis of other organs |  |  |  | 0 |  |
| A20 | Plague |  |  |  | 0 |  |
| A21 | Tularemia |  |  |  | 1 |  |
| A22 | Anthrax |  |  |  | 0 |  |
| A23 | Brucellosis |  |  |  | 0 |  |
| A24 | Glanders and melioidosis |  |  |  | 0 |  |
| A25 | Rat-bite fevers |  |  |  | 0 |  |
| A26 | Erysipeloid |  |  |  | 0 |  |
| A27 | Leptospirosis |  |  |  | 0 |  |
| A28 | Other zoonotic bacterial diseases, not elsewhere classified |  |  |  | 1 |  |
| A30 | Leprosy |  |  |  | 0 |  |
| A31 | Infection due to other mycobacteria |  |  |  | 0 |  |
| A32 | Listeriosis |  |  |  | 0 |  |
| A35 | Other tetanus |  |  |  | 0 |  |
| A39 | Meningococcal infection |  |  |  | 0 |  |
| A40 | Streptococcal sepsis |  |  |  | 1 |  |
| A41 | Other sepsis |  |  |  | 1 |  |
| A42 | Actinomycosis |  |  |  | 0 |  |
| A43 | Nocardiosis |  |  |  | 0 |  |
| A44 | Bartonellosis |  |  |  | 0 |  |
| A48 | Other bacterial diseases, not elsewhere classified |  |  |  | 0 |  |
| A49 | Bacterial infection of unspecified site |  |  |  | 64 |  |
| A51 | Early syphilis |  |  |  | 0 |  |
| A52 | Late syphilis |  |  |  | 0 |  |
| A53 | Other and unspecified syphilis |  |  |  | 0 |  |
| A54 | Gonococcal infection |  |  |  | 8 |  |
| A56 | Other sexually transmitted chlamydial diseases |  |  |  | 0 |  |
| A57 | Chancroid |  |  |  | 0 |  |
| A58 | Granuloma inguinale |  |  |  | 0 |  |
| A65 | Nonvenereal syphilis |  |  |  | 0 |  |
| A66 | Yaws |  |  |  | 0 |  |
| A67 | Pinta |  |  |  | 0 |  |
| A68 | Relapsing fevers |  |  |  | 0 |  |
| A69 | Other spirochaetal infections |  |  |  | 7 |  |
| A70 | Chlamydia psittaci infection |  |  |  | 0 |  |
| A71 | Trahcoma |  |  |  | 0 |  |
| A74 | Other diseases caused by chlamydiae |  |  |  | 0 |  |
| A75 | Typhus fever |  |  |  | 0 |  |
| A77 | Spotted fever [tick-borne rickettsioses] |  |  |  | 0 |  |
| A78 | Q fever |  |  |  | 0 |  |
| A79 | Other rickettsioses |  |  |  | 0 |  |
| B95 | Streptococcus and staphylococcus as the cause of diseases classified to other chapters |  |  |  | 31 |  |
| B96 | Other specified bacterial agents as the cause of diseases classified to other chapters |  |  |  | 0 |  |
| B98 | Other specified infectious agents as the cause of diseases classified to other chapters |  |  |  | 0 |  |
| D73.3 | Abscess of spleen |  |  |  | 0 |  |
| E06.0 | Acute thyroiditis |  |  |  | 0 |  |
| E32.1 | Abscess of thymus |  |  |  | 0 |  |
| G00 | Bacterial meningitis, not elsewhere classified |  |  |  | 0 |  |
| G06 | Intracranial and intraspinal abscess and granuloma |  |  |  | 0 |  |
| H00 | Hordeolum and chalazion |  |  |  | 4 |  |
| H05.0 | Acute inflammation of orbit |  |  |  | 3 |  |
| H44.0 | Purulent endophthalmitis |  |  |  | 0 |  |
| I30 | Acute pericarditis |  |  |  | 1 |  |
| I33 | Acute and subacute endocarditis |  |  |  | 0 |  |
| I40 | Acute myocarditis |  |  |  | 0 |  |
| J34.0 | Abscess, furuncle and carbuncle of nose |  |  |  | 1 |  |
| K11.3 | Abscess of salivary gland |  |  |  | 0 |  |
| K61 | Abscess of anal and rectal regions |  |  |  | 0 |  |
| K65 | Peritonitis |  |  |  | 0 |  |
| M00 | Pyogenic arthritis |  |  |  | 0 |  |
| M60.0 | Infective myositis |  |  |  | 0 |  |
| M65.0 | Abscess of tendon sheath |  |  |  | 0 |  |
| M65.1 | Other infective (teno)synovitis |  |  |  | 0 |  |
| M71.0 | Abscess of bursa |  |  |  | 0 |  |
| M71.1 | Other infective bursitis |  |  |  | 0 |  |
| M72.6 | Necrotizing fasciitis |  |  |  | 0 |  |
| N61 | Inflammatory disorders of breast |  |  |  | 0 |  |
|  |  |  |  |  |  |  |
| **11.6b** | **Other bacterial diseases (respiratory tract)** | **110** | **4** | **3** | **117** | **0.1** |
| A15 | Respiratory tuberculosis, bacteriologically and histologically confirmed |  |  |  | 0 |  |
| A16 | Respiratory tuberculosis, not confirmed bacteriologically or histologically |  |  |  | 1 |  |
| A19 | Miliary tuberculosis |  |  |  | 0 |  |
| A36 | Diphteria |  |  |  | 0 |  |
| A37 | Whooping cough |  |  |  | 16 |  |
| A38 | Scarlet fever |  |  |  | 100 |  |
| J39.0 | Retropharyngeal and parapharyngeal abscess |  |  |  | 0 |  |
| J39.1 | Other abscess of pharynx |  |  |  | 0 |  |
|  |  |  |  |  |  |  |
| **11.7.** | **Diseases of oral cavity** | **12** | **0** | **1** | **13** | **<0.1** |
| K02 | Dental caries |  |  |  | 5 |  |
| K04 | Diseases of pulp and periapical tissues **(excluding the non-infective K04.1, K04.2, K04.3, K04.8, K04.9)** |  |  |  | 2 |  |
| K05 | Gingivitis and periodontal diseases |  |  |  | 5 |  |
| K12.2 | Cellulitis and abscess of mouth |  |  |  | 1 |  |
|  |  |  |  |  |  |  |
| **11.8.** | **Lymphadenitis** | **35** | **4** | **4** | **43** | **<0.1** |
| I88 | Nonspecific lymphadenitis |  |  |  | 3 |  |
| L04 | Acute lymphadenitis |  |  |  | 25 |  |
| R59 | Enlarged lymph nodes |  |  |  | 15 |  |
|  |  |  |  |  |  |  |
| **11.9.** | **Infections related to pregnancy, childbirth and puerperium** | **0** | **0** | **0** | **0** | **0** |
| A34 | Obstetrical tetanus |  |  |  | 0 |  |
| O07.0 | Failed medical abortion, complicated by genital tract and pelvic infection |  |  |  | 0 |  |
| O07.5 | Other and unspecified failed attempted abortion, complicated by genital tract and pelvic infection |  |  |  | 0 |  |
| O08.0 | Genital tract and pelvic infection following abortion and ectopic and molar pregnancy |  |  |  | 0 |  |
| O23 | Infections of genitourinary tract in pregnancy |  |  |  | 0 |  |
| O41.1 | Infection of amniotic sac and membranes |  |  |  | 0 |  |
| O75.3 | Other infection during labour |  |  |  | 0 |  |
| O85 | Puerperal sepsis |  |  |  | 0 |  |
| O86 | Other puerperal infections |  |  |  | 0 |  |
| O91 | Infections of breast associated with childbirth |  |  |  | 0 |  |
| O98 | Maternal infectious and parasitic diseases classifiable elsewhere but conmplicating pregnancy, childbirth and the puerperius |  |  |  | 0 |  |
|  |  |  |  |  |  |  |
| **11.10.** | **Perinatal/neonatal infections** | **2** | **0** | **1** | **3** | **<0.1** |
| A33 | Tetanus neonatorum |  |  |  | 0 |  |
| A50 | Congenital syphilis |  |  |  | 0 |  |
| P23 | Congenital pneumonia |  |  |  | 0 |  |
| P35 | Congenital viral diseases |  |  |  | 0 |  |
| P36 | Bacterial sepsis of newborn |  |  |  | 0 |  |
| P37 | Other congenital infections and parasitic diseases |  |  |  | 0 |  |
| P38 | Omphalitis of newborn with or without mild haemorrhage |  |  |  | 2 |  |
| P39 | Other infections specific to the perinal period |  |  |  | 1 |  |
|  |  |  |  |  |  |  |
| **11.11.** | **Other and unspecified infections** | **3** | **4** | **1** | **8** | **<0.1** |
| A63 | Other predominantly sexually transmitted diseases, not elsewhere classified |  |  |  | 0 |  |
| A64 | Unspecified sexually transmitted disease |  |  |  | 0 |  |
| B99 | Other and unspecified infectious diseases |  |  |  | 8 |  |
| G03 | Meningitis due to other and unspecified causes |  |  |  | 0 |  |
| G04 | Encephalitis, myelitis and encephalomyelitis |  |  |  | 0 |  |
| H83.0 | Labyrinthitis |  |  |  | 0 |  |
